# Dissipation behavior and dietary exposure risk of pesticides in Brussels sprout evaluated using LC–MS/MS

**DOI:** 10.1038/s41598-022-17116-z

**Published:** 2022-07-26

**Authors:** Dai An, Rakdo Ko, Jinchan Kim, Seokhyun Kang, Kwanghun Lee, Jiho Lee

**Affiliations:** 1 Korea Conformity Laboratories, Incheon, 21999 Republic of Korea; 2 Department of Crop Sciences, Konkuk University, Seoul, 05029 Republic of Korea

**Keywords:** Risk factors, Environmental chemistry

## Abstract

In this study, the dissipation behavior and dietary exposure risk of eight pesticides in Brussels sprout were evaluated under greenhouse conditions. Brussels sprout samples were collected 0, 7, 14, and 21 days after the last pesticide treatment. Ultra-high performance liquid chromatography with tandem mass spectrometry was used for sample analysis. Recovery rates at different concentrations of pesticides (0.01 and 0.1 mg/kg) were in the range of 70.2–104.5%, and the relative standard deviations were ≤ 10.6%. The pesticide residues in Brussels sprouts were determined for each treatment. For acephate, etofenprox, imidacloprid, indoxacarb, alpha-cypermethrin, zeta-cypermethrin, fludioxonil, and oxytetracycline, the half-lives were, respectively, 11.3, 9.8, 11.3, 15.8, 10.6, 13, 9.1, and 8.2 d and the dietary intake rates were, respectively, 2.90%, 0.81%, 0.7%, 1.19%, 0.06%, 0.24%, 0.05%, and 0.36% of the acceptable daily intake. The findings of this study provide important insights into the establishment of maximum residue limits in the Republic of Korea and pesticide control measures for Brussels sprout.

## Introduction

Pesticides are widely used to protect crops against pests and diseases and, thus, increase agricultural productivity^1,2^. Pesticide residues may remain on crops; however, pesticide retention poses potential risks to human health and the environment^3,4^. A positive list system has been in place in the Republic of Korea (ROK) since 2019^5^, which regulates the limits for pesticides (0.01 mg/kg) applied to agricultural products for which there is no maximum residue limit (MRL). Although agricultural products must be safer for consumers^6^, maintaining these strict standards is challenging for producers. Pesticide manufacturers have focused on registering their pesticide products for major crops over minor crops^7^ because of the economic importance of the former. Hence, minor crops with cultivation areas of < 1000 ha^8^ have garnered less attention.

Brussels sprout is a minor crop that belongs to the cruciferous group similar to broccoli, cauliflower, and cabbage. The consumption of these vegetables has been associated with a lower occurrence of cancer in humans^9^. Brussels sprout contains several compounds that are beneficial to human health, such as vitamins, minerals, enzymes, and amino acids^10–13^. Brussels sprout exports totaled USD 218.1 million in 2020, representing a 6% increase from the value noted in 2019^14^.

In the European Union (EU), MRLs have been established for several pesticides used for Brussels sprout^15^. However, in the ROK, the MRLs established for cabbage are used for Brussels sprout as well; these MRLs were established without considering the latter species^16^. The MRLs for Brussels sprout (EU) and cabbage (ROK) are 0.01 and 5 mg/kg (acephate), 0.01 and 0.2 mg/kg (etofenprox), 0.5 and 0.5 mg/kg (imidacloprid), 0.06and 0.2 mg/kg (indoxacarb), 1 and 1 mg/kg (alpha-cypermethrin), 1 and 1 mg/kg (zeta-cypermethrin), and 0.01 and 2 mg/kg (fludioxonil), respectively. An MRL for oxytetracycline has not been established for Brussels sprout in either location.

The dissipation patterns of pesticides have been studied in many crops. Previous studies have examined the dissipation of acephate residues in brinjal^17^, green chili fruit^18^, and mango^19^. In the case of etofenprox, there are studies on pesticides’ dissipation patterns in spring onion^20^, tomato^21^, and squash^22^. The dissipation behavior of imidacloprid has been investigated in mango^23^ and grape^24^. Some previous studies have assessed the dissipation behavior of indoxacarb in cabbage^25^, tomato^26^, and green chili fruit^27^. Bae et al.^28^ and Hwang et al.^29^ conducted studies on the half-life of fludioxonil in mandarin and Chinese cabbage, respectively. However, to the best of our knowledge, no studies have assessed the dissipation behavior of the aforementioned eight pesticide residues in Brussels sprout yet. To fill this knowledge gap, the study evaluated the residue patterns, half-lives, MRLs, and dietary risks of pesticide residues in Brussels sprout in greenhouse condition. The findings are expected to contribute toward the establishment of a set of MRLs for pesticide use in Brussels sprout in the ROK.

## Materials and methods

### Chemicals, reagents, and materials

Acephate, methamidophos, etofenprox, fludioxonil, indoxacarb, and imidacloprid were purchased from Kemidas Co. (Suwon, ROK). Alpha-cypermethrin and zeta-cypermethrin (> 99.91% purity) were obtained from HPC Standards GmbH (Borsdorf, Germany). Oxytetracycline was provided by ChemScene LLC (Monmouth Junction, NJ, USA). The physicochemical properties of the pesticides are presented in Supplementary Table S1. Pesticide products were purchased at a local pesticide market (Seoul, ROK; Table 1). High performance liquid chromatography-grade methanol and acetonitrile were provided by J. T. Baker Chemicals (Phillipsburg, NJ, USA). Formic acid (> 98% purity) and ammonium formate (> 98% purity) were obtained from Merck GaA (Darmstadt, Germany) and Sigma-Aldrich Co., Ltd. (St. Louis, MO, USA), respectively. Membrane filters (0.2 and 0.45 µm) were purchased from Phenomenex (Torrance, CA, USA). An EN-QuEChERS kit was purchased from Chiral Technology Korea (Daejeon, ROK).

**Table 1:** Pesticide treatment of Brussels sprout.

Pesticide	Formulation	Active ingredient contents (%)	Treatment times	Treated day before harvest	Dilution ratio	Total spraying amount (L)
Acephate	WP	50	2	30/21	800	14.5
				21/14		14
				14/7		14.5
				7/0		15
Etofenprox + Indoxacarb	WP	10 + 1.5	2	30/21	1000	14
				21/14		15
				14/7		14.5
				7/0		14.5
Imidacloprid	WP	10	2	30/21	2000	14.5
				21/14		14
				14/7		14.5
				7/0		15
Indoxacarb	WP	10	2	30/21	2000	14.5
				21/14		14
				14/7		14.5
				7/0		15
Alpha-cypermethrin	EC	2	2	30/21	1000	14
				21/14		14.5
				14/7		14.5
				7/0		14.5
Zeta-cypermethrin	EC	3	2	30/21	1000	14.5
				21/14		14.5
				14/7		14.5
				7/0		14.5
Fludioxonil	FL	20	2	30/21	2000	14
			3	30/21/14		22
			3	21/14/7		21.5
			3			
				14/7/0		22
Oxytetracycline	WP	17	2	30/21	1000	14.5
			3	30/21/14		21.5
			3	21/14/7		22
			3	14/7/0		21.5

### Field experiments

**Figure 1:**
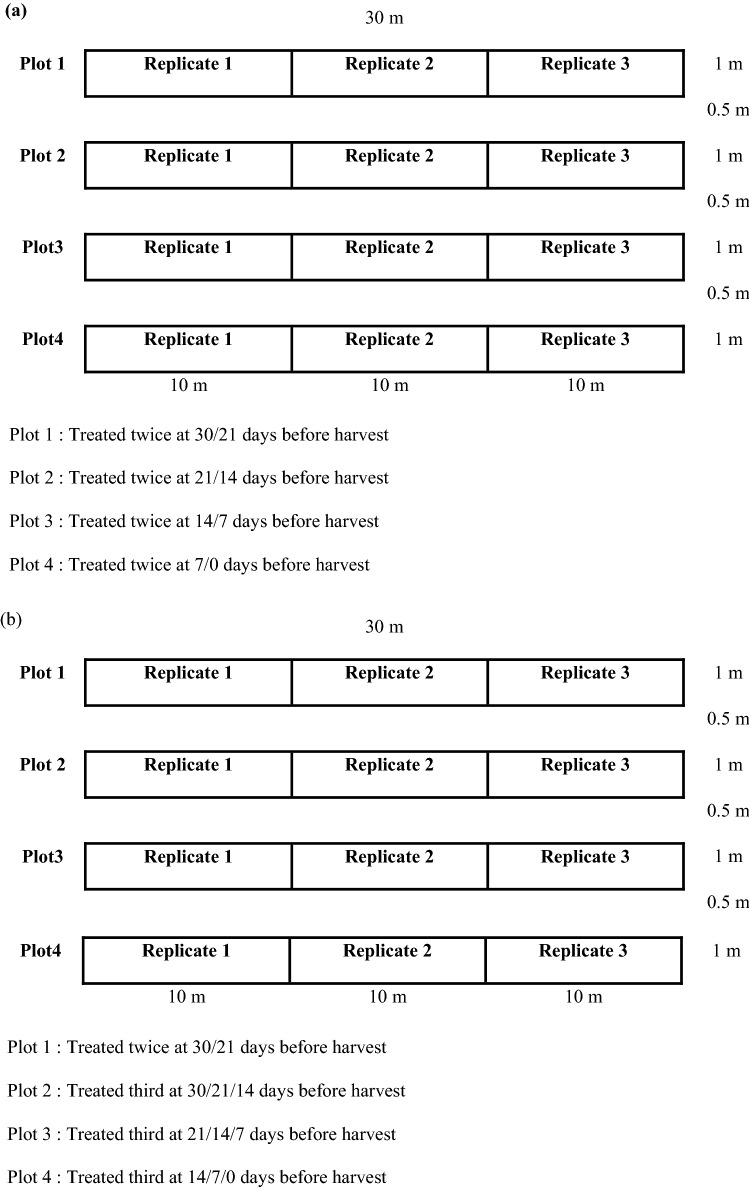
Experimental plots for pesticides treatment of Brussels sprout. (**a**) acephate, etofenprox, imidacloprid, indoxacarb, alpha-cypermethrin, and zeta-cypermethrin (**b**) fludioxonil and oxytetracycline.

The test field was located in Eumseong-gun (GPS coordinates; 36.947296, 127.469195, Chungcheongbuk-do, ROK). The total field size for each pesticide treatment was 30 m (length) × 5.5 m (width). To eliminate the variation in plant growth and climate change, each field was divided into four plots, and samples on each field were harvest on the same day (Table 1). A control plot that was located in an area that was separated from the treated areas. In the cases of acephate, etofenprox, imidacloprid, indoxacarb, alpha-cypermethrin, and zeta-cypermethrin, each plot was treated with pesticide twice before harvest (Fig. 1a). For fludioxonil and oxytetracycline, each plot was treated with the pesticide either twice or thrice before harvest (Fig. 1b). Each plot consisted of 3 replicates, and to eliminate cross-contamination, a buffer zone of 1 m was set for each plot. Information regarding the pesticide treatments is presented in Table 1. Brussels sprout samples (> 1 kg) were collected randomly from each replicate (1/3 plot) at 0, 7, 14, and 21 day after the last pesticide treatment. During Brussels sprout cultivation, the greenhouse air temperature range was − 3.0 to 40.4 °C and humidity was 74.8–98.0% (Supplementary Table S2). All field experiments were performed with the verified organization that is certified the license to conduct the field experiments and collect samples by government (rural development administration in South Korea). All the plant experiments were in compliance with relevant institutional, national, good agricultural practice and international Good Labolatory practice guidelines and legislation^30^.

### Standard solutions

For use in liquid chromatography with tandem mass spectrometry (LC–MS/MS), a standard stock solution of oxytetracycline was prepared at a concentration of 1000 mg/L with methanol. The working solutions were prepared via serial dilution of the stock solution with methanol. Standard stock solutions of the other pesticides were prepared at a concentration of 1,000 mg/L with acetonitrile. The working solutions were prepared via serial dilution of the stock solution with acetonitrile. Each of the standard solutions was mixed with an extract of Brussels sprout (1:1, v/v).

### Sample preparation and extraction

All plant samples were rapidly transferred to the laboratory after harvest. Brussels sprout samples were homogenized with dry ice and then stored at − 20 °C in polyethylene bags.

#### Oxytetracycline

Homogenized Brussels sprout samples (10 g) were weighed in 50-ml falcon tubes. Of the mixture containing 50:50:0.9 volume methanol:water: formic acid, 10 mL was added to the falcon tube. The tube was shaken (1200 rpm) for 1 min. After centrifugation (4000 rpm, 4 °C, 10 min), 1 ml of the supernatant was filtered using a 0.45-μm polytetrafluoroethylene syringe filter.

#### Other pesticides

The EN-QuEChERS kit was used to extract the pesticides^31^. Homogenized Brussels sprout (10 g) was weighed in a 50-mL falcon tube. A total of 10 mL of acetonitrile was added to the tube. The QuEChERS kit components (4 g ofMgSO_4_, 1 g of NaCl, 1 g of sodium citrate, and 0.5 g of disodium citrate sesquihydrate) were added to the falcon tube. The tube was shaken (1200 rpm) for 1 min. After centrifugation (4000 rpm, 5 min), 1 ml of the supernatant was filtered using a 0.2-μm polytetrafluoroethylene syringe filter.

### LC–MS/MS analytical conditions

All samples were analyzed using Shimadzu LC–MS-8045 with UHPLC Nexera X2 (Kyoto, Japan). Chromatographic separation was performed using a Kinetex C18 column (2.1 × 150 mm; 2.6 μm particle size; Phenomenex, USA) maintained at 40 °C. Information regarding the mobile phase, gradient program, and injection volume for all analytes are presented in Supplementary Tables S3a and S3b. A triple quadrupole (QqQ) mass spectrometer (Shimadzu) with a positive electrospray ionization (ESI) source was used for all the analytes, except fludioxonil (negative ESI; Supplementary Table S4). Supplementary Table S4 shows the multi reaction monitoring conditions for all analytes.

### Method performance

The method was validated in terms of linearity, accuracy, precision, and limit of quantitation (LOQ). Linearity was assessed using a pure standard solution and homogenized pesticide-free Brussels sprout based on the data obtained using five concentrations: 5, 10, 20, 50, and 100 ng/mL. The lowest concentration among the chromatograms that produced a signal-to-noise ratio of > 10 was selected as the LOQ. To determine the recovery rate, the pesticide standards were added to the homogenized pesticide-free samples at two different concentrations: 0.01 and 0.1 mg/kg. Each test was performed in triplicate.

### Definition of pesticide residues in Brussels sprout

The individual compounds were analyzed for eight pesticides. The information regarding the residue definitions was provided by ministry of drug and food safety^16^. The parent substance only was evaluated except for acephate residues. Acephate residue in plants is the sum of acephate and methamidophos, which is a metabolite of acephate. The molecular weights of acephate and methamidophos are 183.2 and 141.1, respectively. To evaluate the total acephate in Brussels sprout, the sum of acephate residue concentration was calculated as follows:

1 $$ C_{sum} = C_{acephate} + C_{methamidophos} \times \frac{183.2}{{141.1}}. $$

### Statistical analysis

The dissipation rate was calculated based on the initial residue concentration (average residue level on plot 4, mg/kg) and that measured 21 day after pesticide treatment. The dissipation rate was calculated as follows:

2 $$ dissipation\,rate\left( \% \right) = \frac{{C_{t} }}{{C_{0} }} \times 100 $$

The dissipation patterns of the eight pesticide residues in Brussels sprout over time were expressed using the following first-order kinetics equation (Eq. 3), and the half-lives were calculated using Eq. (4)^32^:

3 $$ C_{t} = C_{0} \times e^{ - kt} $$

4 $$ t_{1/2} = ln\frac{2}{k} $$

where *C*_0_is the initial residue concentration (mg/kg) in plot 4, *C*_*t*_ is the residue concentration (mg/kg) in plots 1, 2, and 3, *t* is the days after pesticide treatment, and *k* is the rate constant of dissipation.

The initial residues were calculated using the MRLs for Brussels sprout in the EU and those for cabbage in the ROK, which is applied to Brussels sprout.

### Dietary risk evaluation

To estimate the human health risk from the pesticide residues present in Brussels sprout, hazard quotient (HQ)was calculated based on the estimated daily intake and ADI using the following Eqs. (5–7):

5 $$ Estimated\,daily\,intake\,\left( { mg d^{ - 1} } \right) = residual\,concentration\,\left( {mg kg^{ - 1} } \right) \times daily\,food\,intake\,\left( {g d^{ - 1} } \right) $$

6 $$ Daily\,intake\,\left( { mg day^{ - 1} } \right) = ADI \left( { mg kg^{ - 1} d^{ - 1} } \right) \times average\,body\,weight\,in\,ROK\,\left( {kg} \right) $$

7 $$ Hazard\,quotient\,\left( {HQ} \right) = \left( {\frac{estimated\,daily\,intake}{{daily\,intake}}} \right) \times 100 $$

In case of the acephate and its metabolite (methamidophos), because their ADIs were different, HQs were calculated separately. The daily dietary intake for Brussels sprout was 7.17 g, which is the daily food intake for vegetables, and the average body weight in the ROK is 59 kg^33^.

## Results and discussion

### LOQ, calibration curve, and recovery

Standard curves of all pesticides showed good linearity in samples of Brussels sprout (Supplementary Table S5). The range was between 0.005 and 0.1 mg/L of standard solution. Method LOQ for all pesticides was 0.01 mg/kg. The accuracy and precision were determined based on the recovery rate and relative standard deviation (RSD) at different concentrations (0.01 and 0.1 mg/kg). Supplementary Table S6 shows the results of the recovery tests. For all pesticides, the range of recovery rates was 70.2–101.9% at low concentrations and 71.5–104.5% at high concentrations. The RSDs for all the pesticides were < 11%.

### Pesticide residues in Brussels sprout

Pesticide residues were not detected in control samples; the pesticide residue results obtained from the field trials are presented in Table 2. The dissipation rate was calculated by comparing the results obtained from plots 1 and 4. Normalization was performed by calculating the residual concentration (mg/kg) compared with the spraying amount (g). The normalized values (NVs) of etofenprox and indoxacarb showed the highest initial concentration values of 1.37 and 1.31, respectively (Table 3). Although oxytetracycline and imidacloprid had low vapor pressure, initial NVs showed the lowest values of 0.24 and 0.45, respectively. The initial NVs were affected by vapor pressure, pesticides’ physicochemical properties, minor substances, and formulation types^20,22,33,34^. For etofenprox, the NV was 1.37 in Brussels sprout; this value has been reported to be 0.06 in squash^22^, 4.37 in squash leaf^22^, 2.09 in Chinese cabbage^34^, and 4.50 in spring onion^20^. The NVs of squash leaf, Chinese cabbage, and spring onion were higher than the value of Brussels sprout because of their surface area and texture. Squash leaves have a large surface area, and the fruit has a slippery surface. The Chinese cabbage studied was the loose-head type vegetable, and spring onion has a greater surface area than the other aforementioned crops; therefore, more pesticides can adsorb to its surface. Regarding indoxacarb, the calculated NVs were 1.31 in Brussels sprout; this value has been reported to be 0.39 in cucumber^35^ as they have different surfaces and properties.

**Table 2:** Average residues and dissipation rates of eight pesticides in Brussels sprout.

Pesticide	Plot (preharvest)	Mean ± SD (mg/kg)	Dissipation rate (%)	EU MRL of Brussels sprout (mg/kg)	Domestic MRL of cabbage (mg/kg)
Acephate	1 (30/21)	1.85 ± 0.48	74.21	0.01	5
	2 (21/14)	4.13 ± 0.53	42.36		
	3 (14/7)	5.18 ± 0.43	27.61		
	4 (7/0)	7.16 ± 1.28	–		
Etofenprox	1 (30/21)	0.43 ± 0.03	78.36	0.01	0.2
	2 (21/14)	0.77 ± 0.07	61.56		
	3 (14/7)	1.12 ± 0.08	43.77		
	4 (7/0)	1.99 ± 0.05	–		
Imidacloprid	1 (30/21)	0.09 ± 0.01	74.26	0.5	0.5
	2 (21/14)	0.24 ± 0.04	27.72		
	3 (14/7)	0.30 ± 0.03	10.89		
	4 (7/0)	0.34 ± 0.04	–		
Indoxacarb	1 (30/21)	0.37 ± 0.10	61.90	0.06	0.2
	2 (21/14)	0.54 ± 0.07	45.24		
	3 (14/7)	0.64 ± 0.02	34.35		
	4 (7/0)	0.98 ± 0.17	–		
Alpha-cypermethrin	1 (30/21)	0.06 ± 0.02	75.18	1	1
	2 (21/14)	0.11 ± 0.02	51.78		
	3 (14/7)	0.17 ± 0.02	29.39		
	4 (7/0)	0.23 ± 0.04	–		
Zeta-cypermethrin	1 (30/21)	0.13 ± 0.03	67.31	1	1
	2 (21/14)	0.23 ± 0.01	43.29		
	3 (14/7)	0.33 ± 0.03	17.51		
	4 (7/0)	0.40 ± 0.04	–		
Fludioxonil	1 (30/21)	0.34 ± 0.19	80.42	0.01	2
	2 (30/21/14)	0.96 ± 0.39	44.53		
	3 (21/14/7)	1.46 ± 0.25	15.93		
	4 (14/7/0)	1.74 ± 0.20	–		
Oxytetracycline	1 (30/21)	0.14 ± 0.03	84.15	–	–
	2 (30/21/14)	0.44 ± 0.03	49.81		
	3 (21/14/7)	0.66 ± 0.13	24.91		
	4 (14/7/0)	0.88 ± 0.05	–		

*SD* standard deviation, *EU* European Union, *MRL* maximum residue limit.

**Table 3:** Normalized values of the eight pesticides.

Pesticide	Initial concentration (mg/kg)	Spraying amount (g)	Normalized value
Acephate	7.16	9.375	0.73
Etofenprox	1.99	1.45	1.37
Imidacloprid	0.34	0.75	0.45
Indoxacarb	0.98	0.75	1.31
Alpha-cypermethrin	0.23	0.29	0.81
Zeta-cypermethrin	0.40	0.435	0.92
Fludioxonil	1.74	2.20	0.79
Oxytetracycline	0.88	3.655	0.24

### Half-lives of pesticides on Brussels sprout

**Figure 2:**
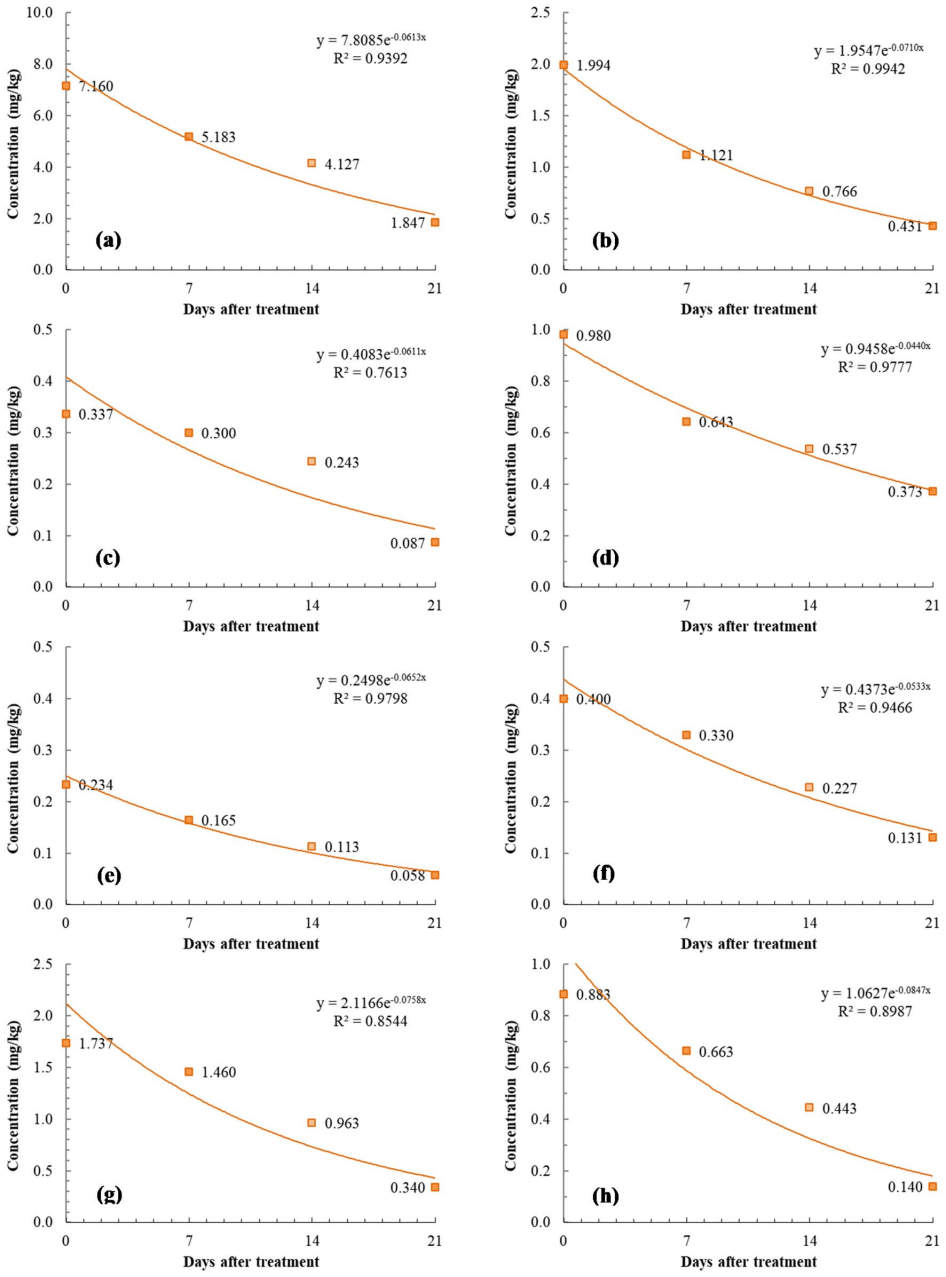
Dissipation patterns of pesticides in Brussels sprout. (**a**) acephate, (**b**) etofenprox, (**c**) imidacloprid, (**d**) indoxacarb, (**e**) alpha-cypermethrin, (**f**) zeta-cypermethrin, (**g**) fludioxonil, and (**h**) oxytetracycline.

The half-lives of the eight pesticides studied vary across crops and are influenced by many factors such as class of target and chemical, plant species, field conditions, chemical and microbial decomposition, dilution by plant growth, volatilization, acidity, photodecomposition, temperature, surface washoff, and spatial variability^36^. Figure 2 shows the regression curves and half-lives of the eight pesticides in Brussels sprout. The half-life for acephate was 11.3 days. Acephate in Brussels sprout decreased slowly compared with acephate in brinjal (2.13 days)^17^, green chili fruit (4.02 days)^18^, and mango (5 days)^19^. The half-life of etofenprox was 9.8 days in Brussels sprout, which is similar to that noted in spring onion (9.5 days)^20^ and higher than that noted in tomato (2.15 days)^21^ and squash (3.5 days)^22^; the differences are attributable to the variations in crop properties and enzymes. Imidacloprid’s half-life (11.3 days) was longer in Brussels sprout than in mango (3.06 days)^23^ and shorter in the former than in grape (16.6 days)^24^. Indoxacarb’s half-life was 15.8 days and decreased slowly compared with indoxacarb’s half-life in cabbage (2.88 days)^25^, tomato (3.21 days)^26^, and green chili fruit (3.85 days)^27^. Hypertrophy—fast crop growth in a short period—is reportedly a major factor in the half-life of pesticides^37,38^. Although cabbage and Brussels sprout have a similar wrapped-over form, the half-lives are different because of the hypertrophy of cabbage. The half-life of fludioxonil in Brussels sprout (9.1 days) was similar to that noted in mandarin (8.7 days)^29^ and longer than that noted in Chinese cabbage (4.0 days)^28^. Mandarins have a bumpy surface, and pesticides are, therefore, likely to adsorb to the surface. The half-lives of alpha-cypermethrin, zeta-cypermethrin, and oxytetracycline were 10.6, 13, and 8.2 days, respectively. In the aspect of chemical class of pesticides, dissipation half-lives range from 0.9 to 22.8 days for organophophates, 0.8–10.6 days for carbamates, 3.1–9.3 days for neonicotinoids, and 1.1–5 days for pyrethroids. Except acephate, most of dissipation half-lives were higher than previous reports^36^. In the aspect of plant species, dissipation half-lives of pesticides were in the range of 0.9–13 days which is similar with this study. In this study, there was less correlation between dissipation half-lives and volatilization (vapor pressure).

### MRLs of pesticides in Brussels sprout

The initial residues (mg/kg) of the eight pesticides were compared with the domestic MRLs for cabbage and that in the EU for Brussels sprout. The initial acephate residue was 143% of the domestic MRL (5 mg/kg) and 71,600% of the EU MRL (0.01 mg/kg). In the case of etofenprox, the initial residue was 1.99 mg/kg, which was 995% of the domestic MRL (0.2 mg/kg) and 19,900% of the EU MRL (0.01 mg/kg). The initial imidacloprid residue (0.34 mg/kg) was 68% of the EU and domestic MRLs (0.5 mg/kg for both). The initial indoxacarb residue (0.98 mg/kg) was 490% of the domestic MRL (0.2 mg/kg) and 1633% of the EU MRL (0.06 mg/kg). The initial residues of alpha-cypermethrin and zeta-cypermethrin were 0.23 and 0.40 mg/kg, respectively; these were 23% and 40% of both MRLs (1 mg/kg). The initial fludioxonil residue (1.74 mg/kg) was 87% of the domestic MRL (2 mg/kg) and 17,400% of the EU MRL (0.01 mg/kg). For oxytetracycline, the initial residue was 0.88 mg/kg but no MRLs have been established in the EU or ROK. The residual amount of oxytetracycline may be used as basic data for the establishment of an MRL. There are no differences between domestic and EU MRLs of imidacloprid, alpha-cypermethrin, and zeta-cypermethrin; however, considerable differences were noted between the EU and ROK MRLs for acephate, etofenprox, indoxacarb, and fludioxonil. The differences highlight the variability in residue patterns with growing conditions, climate, and crop species.

### Dietary risk assessment

In Brussels sprout, residues for all eight pesticides of the %ADI values for ROK average intake were < 3% (2.90%, 0.81%, 0.7%, 1.19%, 0.06%, 0.24%, 0.05%, and 0.36% for acephate, etofenprox, imidacloprid, indoxacarb, alpha-cypermethrin, zeta-cypermethrin, fludioxonil, and oxytetracycline, respectively; Table 4). Considering the daily consumption of Brussels sprout, the exposure risk to pesticides is considered to be low.

**Table 4:** Estimated daily intake of pesticides via the dietary intake of Brussels sprout.

Pesticide	Initial concentration (mg/kg)	Estimated daily intake (mg/kg)	Acceptable daily intake (mg/kg/)	HQ
Acephate	6.82	0.049	0.03	2.76 × 10^−2^
Methamidophos	0.33	0.002	0.004	1.00 × 10^−2^
Etofenprox	1.99	0.014	0.03	8.08 × 10^−3^
Imidacloprid	0.34	0.002	0.06	5.28 × 10^−4^
Indoxacarb	0.98	0.007	0.01	6.82 × 10^−4^
Alpha-Cypermethrin	0.23	0.002	0.05	1.19 × 10^−2^
Zeta-Cypermethrin	0.40	0.003	0.02	5.68 × 10^−4^
Fludioxonil	1.74	0.012	0.40	2.43 × 10^−3^
Oxytetracycline	0.88	0.006	0.03	3.58 × 10^−3^

## Conclusions

We evaluated the residue, dissipation pattern, and dietary risk of acephate, etofenprox, imidacloprid, indoxacarb, alpha-cypermethrin, zeta-cypermethrin, fludioxonil, and oxytetracycline in Brussels sprout. The half-lives of the pesticides in Brussels sprout were determined to be 11.3 (acephate), 9.8 (etofenprox), 11.3 (imidacloprid), 15.8 (indoxacarb), 10.6 (alpha-cypermethrin), 13 (zeta-cypermethrin), 9.1 (fludioxonil), and 8.2 (oxytetracycline) days. Pesticide residue is affected by various factors such as vapor pressure, pesticides’ physicochemical properties, minor substances, formulation types, environment conditions, crop species, and growth dilution factors. Based on the %ADI values, it can be concluded that the intake of pesticide residues from Brussels sprout does not pose a significant health risk. These findings provide useful information for the establishment of MRLs in the ROK and pesticide control measures for Brussels sprout.

## Supplementary Information

Supplementary Information.

## Data Availability

The data that support the findings of this study are available from the corresponding author upon reasonable request.
