# Supplementary material for: Dissipation behavior and dietary exposure risk of pesticides in Brussels sprout evaluated using LC–MS/MS
Source: Sci Rep. 2022 Jul 26;12:12726. doi: 10.1038/s41598-022-17116-z (PMC9325977; doi:10.1038/s41598-022-17116-z)
Supplement: Supplementary file 1 — Supplementary Information. [file 41598_2022_17116_MOESM1_ESM.docx]

Supplementary Table S1. Chemical structures and physicochemical properties of eight pesticides

| **Pesticide** | **Chemical structure** | **Molecular weight** | **Vapor pressure(mPa)** |
| --- | --- | --- | --- |
| Acephate | 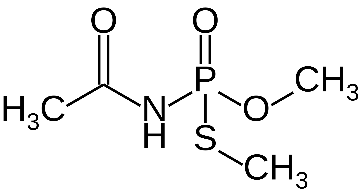 | 183.2 | 0.226 (24°C) |
| Etofenprox | 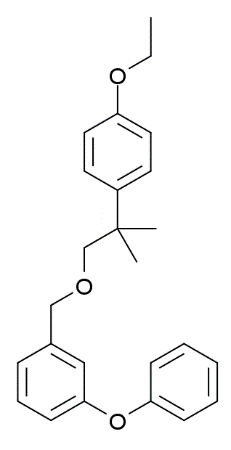 | 376.5 | 8.13 × 10^−4^(25°C) |
| Imidacloprid | 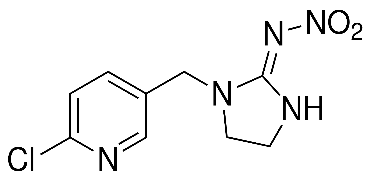 | 255.7 | 9 × 10^−7^ (25°C) |
| Indoxacarb | 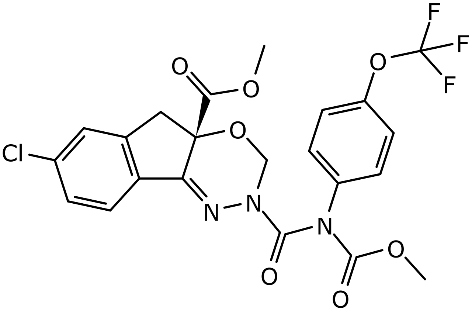 | 527.8 | 2.5 × 10^−5^ (25°C) |
| Alpha-cypermethrin | 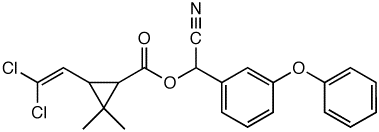 | 416.3 | 2.3 × 10^−2^ (20°C) |
| Zeta-cypermethrin | 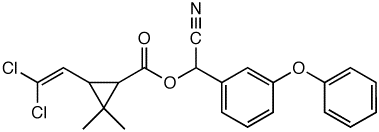 | 416.3 | 2.5 × 10^−4^ (25°C) |
| Fludioxonil | 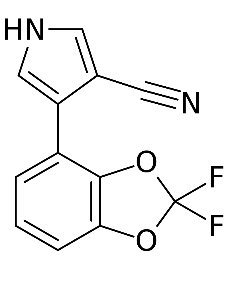 | 248.2 | 3.9 × 10^−4^ (25°C) |
| Oxytetracycline | 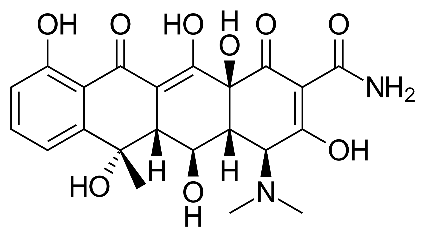 | 460.4 | 1.29 × 10^−19^ (25°C) |

Supplementary Table S2. During cultivation of squash fruit and leaf, the greenhouse air temperature, humidity, and pesticide treatment date

| **Date** | **Temperature (℃)** | | | **Average Humidity (%)** | **Pesticide treatment date** |
| --- | --- | --- | --- | --- | --- |
|  | **Minimum** | **Maximum** | **Average** |  |  |
| 25-Oct-21 | 2.2 | 32.5 | 12.1 | 79.1 | pesticides treatment 30 days before harvest |
| 26-Oct-21 | 2.5 | 29.8 | 12.5 | 82.0 |  |
| 27-Oct-21 | 37. | 39.6 | 14.5 | 81.5 |  |
| 28-Oct-21 | 1.3 | 29.7 | 11.- | 75.3 |  |
| 29-Oct-21 | 2.7 | 31.1 | 12.6 | 74.8 |  |
| 30-Oct-21 | 5.3 | 28.6 | 13.7 | 90.7 |  |
| 31-Oct-21 | 4.0 | 40.4 | 15.4 | 87.0 |  |
| 1-Nov-21 | 5.1 | 29.1 | 12.9 | 87.5 |  |
| 2-Nov-21 | 4.4 | 28.9 | 12.2 | 83.7 |  |
| 3-Nov-21 | 2.8 | 38.5 | 14.3 | 88.3 | pesticides treatment 21 days before harvest |
| 4-Nov-21 | 6.0 | 36.4 | 14.7 | 93.7 |  |
| 5-Nov-21 | 5.3 | 30.9 | 13.1 | 87.5 |  |
| 6-Nov-21 | 2.7 | 39.6 | 15.2 | 88.9 |  |
| 7-Nov-21 | 3.3 | 38.9 | 15.9 | 90.4 |  |
| 8-Nov-21 | 7.3 | 17.1 | 10.7 | 97.7 |  |
| 9-Nov-21 | 6.2 | 31.9 | 10.1 | 96.1 |  |
| 10-Nov-21 | 0.5 | 16.7 | 7.0 | 97.1 | pesticides treatment 14 days before harvest |
| 11-Nov-21 | 0.1 | 30.6 | 9.2 | 94.4 |  |
| 12-Nov-21 | 0.7 | 35.1 | 10.5 | 92.0 |  |
| 13-Nov-21 | -1.8 | 33.5 | 10.0 | 92.5 |  |
| 14-Nov-21 | 2.9 | 31.5 | 12.2 | 94.9 |  |
| 15-Nov-21 | 0.7 | 36.1 | 11.5 | 93.8 |  |
| 16-Nov-21 | 0.6 | 35.6 | 11.3 | 93.4 |  |
| 17-Nov-21 | -1.1 | 35.6 | 10.4 | 92.5 | pesticides treatment 7 days before harvest |
| 18-Nov-21 | 1.9 | 27.0 | 10.1 | 86.3 |  |
| 19-Nov-21 | 3.3 | 20.6 | 10.5 | 90.0 |  |
| 20-Nov-21 | 4.2 | 24.5 | 10.4 | 98.0 |  |
| 21-Nov-21 | 6.5 | 24.5 | 11.0 | 97.9 |  |
| 22-Nov-21 | 1.0 | 27.3 | 9.5 | 94.4 |  |
| 23-Nov-21 | -3.0 | 17.1 | 4.2 | 94.4 |  |
| 24-Nov-21 | -1.9 | 13.5 | 4.8 | 96.3 | pesticides treatment 0 day before harvest and sampling |

Supplementary Table S3a. LC-MS/MS analytical conditions for six pesticides (acephate, etofenprox, fludioxonil, imidacloprid, indoxacarb, and oxytetracycline)

| **LC** | **Condition** | | | | | | | | | | | |
| --- | --- | --- | --- | --- | --- | --- | --- | --- | --- | --- | --- | --- |
| System | Shimadzu LCMS-8045 with UHPLC Nexera X2 | | | | | | | | | | | |
| Column | Kinetex C18, 2.1 × 150 mm, 2.6 μm | | | | | | | | | | | |
| Mobile phase | A: 0.1% formic acid in water  B: 0.1% formic acid in acetonitrile | | | | | | | | | | | |
|  | Acephate | | Etofenprox | | Imidacloprid | | Indoxacarb | | Fludioxonil | | Oxytetracycline | |
| Flow rate (ml/min) | 0.2 | | 0.2 | | 0.2 | | 0.2 | | 0.2 | | 0.3 | |
| Injection volume (μl) | 2 | | 2 | | 2 | | 2 | | 50 | | 20 | |
| Gradient | Time | B conc. | Time | B conc. | Time | B conc. | Time | B conc. | Time | B conc. | Time | B conc. |
|  | 0 | 0 | 0 | 10 | 0 | 20 | 0 | 10 | 0 | 20 | 0 | 5 |
|  | 1 | 0 | 1 | 10 | 1 | 20 | 1 | 10 | 1 | 20 | 2 | 5 |
|  | 2 | 90 | 2 | 90 | 2 | 90 | 2 | 90 | 2 | 90 | 2.5 | 50 |
|  | 5 | 90 | 5 | 90 | 5 | 90 | 5 | 90 | 5 | 90 | 4 | 50 |
|  | 6 | 0 | 6 | 10 | 6 | 20 | 6 | 10 | 6 | 20 | 4.5 | 90 |
|  | 7 | 0 | 7 | 10 | 7 | 20 | 7 | 10 | 7 | 20 | 5.5 | 90 |
|  |  |  |  |  |  |  |  |  |  |  | 6 | 5 |
|  |  |  |  |  |  |  |  |  |  |  | 7 | 5 |
| **MS** | **Condition** | | | | | | | | | | | |
| Ionization | Electrospray ionization (ESI) | | | | | | | | | | | |
|  | Acephate | | Etofenprox | | Imidacloprid | | Indoxacarb | | Fludioxonil | | Oxytetracycline | |
| Interface temperature | 250 | | 300 | | 350 | | 300 | | 350 | | 350 | |
| Dl temperature | 200 | | 250 | | 300 | | 250 | | 300 | | 300 | |
| Heat block temperature | 400 | | 400 | | 400 | | 400 | | 400 | | 400 | |

Supplementary Table S3b. LC-MS/MS analytical conditions for alpha- and zeta-cypermethrin

| **LC** | **Condition** | | | | |
| --- | --- | --- | --- | --- | --- |
| System | Shimadzu LCMS-8045 with UHPLC Nexera X2 | | | | |
| Column | Kinetex C18, 2.1 × 150 mm, 2.6 μm | | | | |
| Mobile phase | A: 5 mM ammonium formate + 0.1% formic acid in water  B: 5 mM ammonium formate + 0.1% formic acid in methanol | | | | |
|  | Alpha-cypermethrin | | | Zeta-cypermethrin | |
| Flow rate (ml/min) | 0.3 | | | 0.2 | |
| Injection volume (μl) | 2 | | | 2 | |
| Gradient | Time | B conc. | | Time | B conc. |
|  | 0 | 5 | | 0 | 60 |
|  | 1 | 5 | | 1 | 60 |
|  | 2 | 100 | | 3.5 | 100 |
|  | 4.5 | 100 | | 5 | 100 |
|  | 5.5 | 5 | | 6 | 60 |
|  | 8 | 5 | | 8 | 60 |
| **MS** | **Condition** | | | | |
| Ionization | Electrospray ionization (ESI) | | | | |
|  | Alpha-cypermethrin | | Zeta-cypermethrin | | |
| Interface temperature | 200 | | 200 | | |
| Dl temperature | 150 | | 150 | | |
| Heat block temperature | 400 | | 400 | | |

Supplementary Table S4. Multiple reaction monitoring conditions of eight analytes

| **Analyte** | **Ionization** | **Precursor ion  (*m/z*)** | **Product ion  (m/z)** | | **Retention time (min)** |
| --- | --- | --- | --- | --- | --- |
|  |  |  | **Quantitation (collision energy, eV)** | **Qualification (collision energy, eV)** |  |
| Acephate | [M + H]^+^ | 183.9 | 49.15 (−22) | 79.15 (−22) | 3.5 |
| Metamidophos | [M + H]^+^ | 141.9 | 47 (−39) | 79 (−29) | 1.3 |
| Etofenprox | [M + NH₄]^+^ | 394.3 | 177.1 (−15) | 359.1 (−12) | 4.8 |
| Imidacloprid | [M + H]^+^ | 256 | 209 (−16) | 175.05 (−19) | 3.3 |
| Indoxacarb | [M + H]^+^ | 527.8 | 150 (−30) | 249.05 (−16) | 3.8 |
| Alpha-cypermethrin | [M + NH₄]^+^ | 433.15 | 190.95 (−17) | 181.05 (−46) | 3.3 |
| Zeta-cypermethrin | [M + NH₄]^+^ | 433.15 | 191.05 (−17) | 126.95 (−32) | 5 |
| Fludioxonil | [M**−** H]^−^ | 247 | 180.15 (27) | 126.15 (32) | 3.8 |
| Oxytetracycline | [M + H]^+^ | 461.1 | 426.15 (−20) | 443.1 (−14) | 3.6 |

Supplementary Table S5. Linear equation of the calibration curves for quantification of the pesticide residues in Brussels sprout

| **Pesticide** | **Linear equation** | **R²** | **point** |
| --- | --- | --- | --- |
| Acephate | y = 639,083.0502 × +492.4543 | 0.9990 | 5 |
| Metamidophos | y = 21,066.2055 × −237.8960 | 0.9930 | 5 |
| Etofenprox | y = 7,270,030.4612 × −8,461.6541 | 0.9965 | 5 |
| Imidacloprid | y = 1,500,137.2573 × +384.9430 | 0.9974 | 5 |
| Indoxacarb | y = 4,198,036.9741 × +5,118.5639 | 0.9991 | 5 |
| Alpha-cypermethrin | y = 2,007,483.3738 × +5,443.0303 | 0.9991 | 5 |
| Zeta-cypermethrin | y = 3,610,339.6845 × +9,630.2633 | 0.9997 | 5 |
| Fludioxonil | y = 81,944.0890 × +5,267.3354 | 0.9991 | 5 |
| Oxytetracycline | y = 349,573.9280 × −12,260.2067 | 0.9991 | 5 |

Supplementary Table S6. Recovery tests

| **Pesticide** | **Fortification (mg/kg)** | | | | | | | | | |
| --- | --- | --- | --- | --- | --- | --- | --- | --- | --- | --- |
|  | **0.01 mg/kg** | | | | | **0.1 mg/kg** | | | | |
|  | **Recovery (%)** | | | | **RSD** | **Recovery (%)** | | | | **RSD** |
|  | **Rep. 1** | **Rep. 2** | **Rep. 3** | **Avg. ± SD** |  | **Rep. 1** | **Rep. 2** | **Rep. 3** | **Avg. ± SD** |  |
| Acephate | 88.5 | 84.6 | 82.5 | 85.2 ± 3.0 | 3.6 | 84.5 | 85.6 | 81.8 | 84.0 ± 2.0 | 2.3 |
| Metamidophos | 99.6 | 98.2 | 99.5 | 99.1 ± 0.8 | 0.8 | 83.0 | 85.8 | 90.3 | 86.4 ± 3.7 | 4.3 |
| Etofenprox | 92.0 | 88.3 | 84.2 | 88.2 ± 3.9 | 4.4 | 87.2 | 85.2 | 102.9 | 91.8 ± 9.7 | 10.6 |
| Imidacloprid | 89.8 | 90.9 | 91.9 | 90.9 ± 1.1 | 1.2 | 88.6 | 87.8 | 88.3 | 88.2 ± 0.4 | 0.5 |
| Indoxacarb | 71.9 | 77.0 | 72.7 | 73.9 ± 2.7 | 3.7 | 79.7 | 71.6 | 80.7 | 77.3 ± 5.0 | 6.5 |
| Alpha-cypermethrin | 73.8 | 71.8 | 72.2 | 72.6 ± 1.1 | 1.5 | 79.6 | 72.9 | 72.1 | 74.9 ± 4.1 | 5.5 |
| Zeta-cypermethrin | 75.4 | 75.9 | 70.2 | 73.8 ± 3.2 | 4.3 | 91.5 | 91.1 | 89.6 | 90.7 ± 1.0 | 1.1 |
| Fludioxonil | 83.6 | 79.8 | 75.3 | 79.6 ± 4.2 | 5.2 | 104.5 | 94.5 | 90.8 | 96.6 ± 7.1 | 7.3 |
| Oxytetracycline | 97.8 | 94.4 | 101.9 | 98.0 ± 3.8 | 3.8 | 75.2 | 73.1 | 71.5 | 73.3 ± 1.9 | 2.5 |

SD, standard deviation; RSD, relative standard deviation.
